# Routinely recorded versus dedicated time registrations during trauma work-up

**DOI:** 10.1186/1752-2897-8-11

**Published:** 2014-08-06

**Authors:** Joanne C Sierink, Evin WM de Jong, Niels WL Schep, J Carel Goslings

**Affiliations:** 1 Trauma Unit, Department of Surgery, Academic Medical Center, Meibergdreef 9, Amsterdam 1105, AZ, the Netherlands

## Abstract

**Introduction:**

Since time intervals are used to determine quality of trauma care, it is relevant to know how reliable those intervals can be measured. The aim of our study was to assess the reliability of time intervals as recorded in our hospital databases.

**Patients and methods:**

We conducted a prospective study on time intervals in our level-1 trauma centre and compared those with the routinely recorded data from February 2012 to June 2012. A convenience sample of all trauma patients admitted to our trauma room was included. The routinely recorded time intervals were retrieved from computerised hospital databases. The dedicated time registration was done on a standardised form on which five time intervals considered clinically relevant were evaluated for each patient by a dedicated person: trauma room time, time to start CT, imaging time, time from trauma room to ICU and time from trauma room to intervention.

**Results:**

In a sample of 100 trauma patients dedicated registered trauma room time was median 47 minutes (IQR = 32-63), compared to 42 minutes (IQR = 28-56) in routinely recorded in hospital databases (P < 0.001). Time to start of CT scanning differed significantly as well, with again an increased time interval measured dedicatedly (median 20 minutes (IQR = 15-28)) compared to the routinely recorded time registration (median 13 minutes (IQR = 4-21)). The other time intervals recorded did not differ between the dedicated and routinely recorded registration. Bland-Altman plots also showed that there is considerable discrepancy between the two measurement methods with wide limits of agreement.

**Conclusion:**

This study shows that routinely recorded time intervals in the trauma care setting differ statistically significant from dedicatedly registered intervals.

## Introduction

Time is one of the important issues in trauma and acute care surgery. Optimal pre-hospital and in-hospital time management can be of life saving importance. Although the Golden Hour concept is based upon an expert opinion rather than solid scientific evidence [1], national trauma databases register time intervals to be able to analyze time-management in the acute trauma care setting [2].

Time intervals are therefore also used as a quality indicator in trauma care [3,4], although there is no high-level evidence to support the correlation between time intervals and quality of care [5,6]. Clearly defined and bases on solid scientific evidence are fundamental prerequisites for useful performance indicators [3]. In the evaluation of trauma care however a wide diversity in quality indicators is used and there is no clear set of broadly accepted indicators [3]. In order to improve performance measurement by means of quality indicators, the American College of Surgeons (ACS) Committee on Trauma has set up a National Surgical Quality Improvement Program (NSQIP) [7]. In the NSQIP, several time intervals, such as time to CT and time to laparotomy or craniotomy, are used as quality indicators [7,8]. If time intervals are used to determine quality of care, it is relevant to know how reliable those intervals can be measured.

In the Dutch Trauma Registry, admission time and time of departure from the trauma room are the only time points that are registered. For quality control, performance improvement and research purposes however, other clinically relevant time points can be retrieved from hospital databases. The reliability and usability of time intervals routinely recorded in several hospital databases is not clear.

Therefore, the aim of our study is to assess the reliability of time intervals as recorded in our hospital databases.

## Patients and methods

We conducted a prospective study on time intervals in our level-1 trauma centre and compared those with the routinely recorded data from February 2012 to June 2012. A convenience sample of all trauma patients admitted to our trauma room was included. All trauma patients admitted to the trauma room during office hours were enrolled (Monday to Friday, 8 am-6 pm). Patients admitted during night and weekend shifts were occasionally enrolled, depending on the availability of the researcher. To assure that the convenience sample taken was representative for the population as whole, baseline characteristics between in- and excluded patients were compared.

The study setting was a level-1 trauma centre in The Netherlands with approximately 750 trauma room admissions each year of which approximately 200 multi trauma patients. Trauma work-up is done according to ATLS® guidelines [9]. Radiologic imaging consists of the standard evaluation with chest and pelvic X-rays, FAST and selective CT scanning. A second trial (REACT-2) was conducted during the study period. Patients included in the REACT-2 trial are randomized between the standard evaluation and an immediate total-body CT scan [10]. A movable 64-slice CT scanner (SOMATOM Sensation 4; Siemens Medical Systems, Erlangen, Germany) is located in the trauma room [11,12].

Time intervals that are routinely recorded as a standard operational procedure (either fully computerised or by nursing staff) are further mentioned ‘routinely recorded’. The routinely recorded time intervals were retrieved from the following databases: admission time and time of departure from the trauma room are routinely registered in the computerised hospital database by nursing staff. Start and end of radiologic imaging and time of arrival at the angiography suite are registered in a radiologic database (acquisition times of images). Time of arrival at the operating room is routinely registered by the OR nursing staff in the computerised operating report and time of arrival at the ICU is routinely registered in the computerised ICU database when a patient is connected to a ventilator or other monitoring device.

The dedicated time registration was registered on a standardized form on which the five time intervals considered clinically relevant were registered. The definitions for starting and stopping the time registration are depicted in Table 1. These definitions are based upon the routinely recorded time registration. The same definitions were used for the dedicated time registration.

**Table 1:** Definitions of starting and stopping time registrations

**Recorded time intervals**	**Start recording**	**Stop recording**
Trauma room time	Patient enters trauma room	Patient leaves trauma room
Time to start CT-scanning	Patient enters trauma room	First CT image obtained
Imaging time	First image obtained during trauma work up	Last image obtained during trauma-workup
Time from admission on Trauma Room to ICU	Patient enters trauma room	Patient arrives at the ICU
Time from admission on Trauma Room to intervention (either angiographical or surgical)	Patient enters trauma room	Patient arrives at angiography suite/OR

The definitions are based upon the routinely recorded time registration. The same definitions were used for the dedicated recorded time registration. Time registration was done by an independent researcher who was not involved in actual trauma care. Recording was started and stopped when the patient crossed the threshold.

Time registration was done by an independent researcher who was not involved in actual trauma care. The researcher was on call during office hours (8 am to 6 pm) and occasionally during weekends and nights. Times were recorded using a smart phone with a stopwatch application. Since the times in the computerised databases are rounded to the minute, the same was done to the times measured with the stopwatch application.

All data were imported in SPSS (version 19.0; SPSS Inc, Chicago, IL). Descriptive statistics were used to describe the data. The Wilcoxon matched-pairs signed-ranks test was used to analyse the time differences between the dedicated and routinely recorded time registration. A p-value less the 0.05 is considered significant. Furthermore, the Bland-Altman [13] plot was used to assess the relative agreement between the dedicated and routinely recorded time measurements. The ‘limits of agreement’ are defined by Bland-Altman as the mean of the difference between the two measurement methods plus or minus 1.96 times the standard deviation of the mean.

## Results

In total, 338 patients were admitted to the trauma room during the study period. The analysed convenience sample consisted of 100 trauma patients (30% of the total population admitted to the trauma room in the study period). There were no statistically significant differences found in age, sex, trauma mechanism, ISS, ICU stay and trauma-related mortality of included patients versus excluded patients, except for the length of total hospital stay (2 days (IQR = 1-7) versus 2 days (IQR = 1-5), P = 0.019).

Characteristics of the convenience sample are depicted in Table 2. Median age was 40 years, the majority of patients were male (68%) sustaining blunt trauma (97%) and median ISS was 5 (IQR = 1-13). There were 20 multi trauma patients in the convenience sample and trauma related mortality was 5%.

**Table 2:** Patient characteristics

	**n = 100**
Age (years)	40.4 (IQR = 22.7-63.3)
Men	68 (68%)
Blunt trauma	97 (97%)
Mechanism of injury	
- fall from height	26 (26%)
- motor vehicle collision	36 (36%)
- bicycle accident	16 (16%)
- penetrating	2 (2%)
- other	20 (20%)
ISS	4.5 (IQR = 1-13)
Multi trauma patients (defined as ISS > 15)	20 (20%)
Hospital stay (days)	2 (IQR = 1-7)
ICU stay (days)	2 (IQR = 1-5)
Ventilation time (days)	2 (IQR = 1-4.8)
Trauma-related mortality	5 (IQR = 5%)

Data are number (%) or median (interquartile range (IQR)). *Abbreviations: ICU* Intensive Care Unit, *ISS* Injury severity score.

The dedicatedly and routinely recorded time registrations are shown in Table 3. Total trauma room time was median 47 minutes (IQR = 32-63) in the dedicated time registration and median 42 minutes (IQR = 28-56) in the routinely recorded time registration (p < 0.001). Time to start CT differed significantly as well, with again an increased time interval measured dedicatedly (median 20 minutes (IQR = 15-28)) compared to the routinely recorded time registration (median 13 minutes (IQR = 4-21)). The other time intervals recorded did not differ between the dedicated and routinely recorded registration.

**Table 3:** Time registration in minutes dedicatedly vs. routinely recorded

	**Dedicated time registration**	**Routinely recorded**	**P-value**
TR time (n = 100)	46.5 (32.3-62.8)	41.5 (28–55.8)	<0.001
Time to start CT-scanning (n = 77)	20 (14.5-27.5)	13 (3.5-21)	<0.001
Imaging time (n = 100)	18 (7.3-25)	18.5 (8–25)	0.180
Time from TR to ICU (n = 21)*	56 (47.8-91.5)	58 (49.5-96)	0.410
Time from TR to intervention (n = 17)*	199 (78–261)	201 (88–256)	0.379

Data are number (%) or median (interquartile range (IQR)). *Abbreviations: TR* trauma room, *ICU* intensive care unit. Trauma Room time is time between arrival at and departure from the trauma room. *Other patients were admitted to the general ward or discharged from the ED.

**Figure 1:**
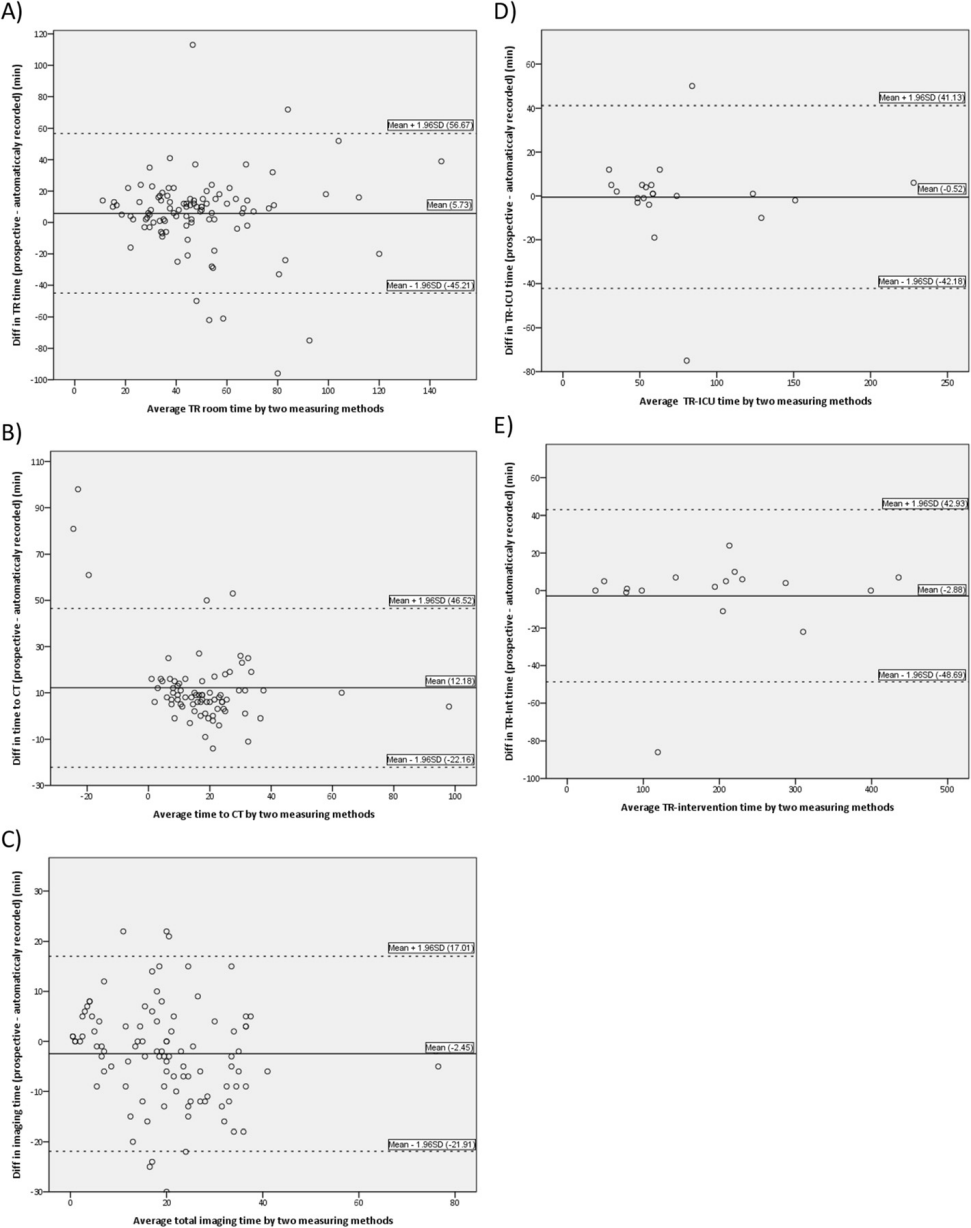
**Bland-Altman plots (difference against mean) for measured time intervals.** Abbreviations: SD, standard deviation; TR, trauma room; ICU, intensive care unit; Int, intervention.

Figure 1 depicts the Bland-Altman plots of the levels of agreement for the two time measurement methods. The plots showed a random nature of the spreads with biases in each plot. However, each time interval shows wide ‘limits of agreement’, reflected by the small sample size and great variation of the differences [13]. For example, the routinely recorded total trauma room time may be 45 minutes below or 57 minutes above the dedicatedly recorded time. Although most observations are within the limits of agreement, we assumed that the wideness of the limits would be relevant for research purposes. This was the case for time to CT as well (routinely recorded time may be 22 minutes below or 47 minutes above the dedicatedly recorded time). The range was less wide in total imaging time with 21 minutes below and 17 minutes above which might be acceptable for research purposes. For the time intervals trauma room to ICU and trauma room to intervention there were wide intervals, but those are difficult to interpret due to the small sample sizes.

Table 4 sets out the time intervals measured according to ISS. Patients with an ISS between 16 and 24 have the longest trauma room time with both measurement methods (52 minutes with the dedicated measurement and 43 minutes with the routinely recorded measurement) while patients with an ISS above 24 have the shortest time at the trauma room (44 minutes with the dedicated measurement and 38 minutes with the routinely recorded measurement).

**Table 4:** Injury severity score versus Trauma Room time (dedicatedly registered) in minutes

**ISS**	**Dedicated time registration**	**Routinely recorded**	**P-value**
1-15 (n = 80)	46 (32–62)	42 (27–56)	0.001
16-24 (n = 11)	53 (40–71)	43 (37–90)	0.756
25-75 (n = 9)	44 (35–53)	38 (28–47)	0.075

Data are number (%) or median (interquartile range (IQR)). *Abbreviations: ISS* injury severity score, *TR* trauma room. Trauma Room time is time between arrival at and departure from the trauma room.

## Discussion

This study shows that routinely recorded time intervals in the trauma care setting differ statistically significant from dedicatedly registered intervals. In a convenience sample of a general trauma population, dedicated registered trauma room time is 47 minutes compared to 42 minutes routinely recorded in hospital databases. Time to start CT is longer when dedicated registered as well. Bland-Altman plots also show that there is considerable discrepancy between the two measurement methods with wide limits of agreement. It depends on the research topic whether wide intervals are acceptable or not.

We believe that most hospitals would argue that time points registered in their hospital databases are in fact dedicatedly collected data. Although this should ideally be the case, we hypothesised that it is well possible that time points retrieved from hospital databases are less prospective and less accurate then we assume. For example, admission and departure times are registered by hand in the medical record by personnel which has other (potentially more important) duties in patient care as well. Therefore we compared those time points with purely dedicatedly collected time points. This dedicated and purely prospective form of data collection is performed in several centres in Germany as well, by using dedicated software to collect data including time intervals for the national trauma registry [14].

Since clinically relevant time intervals in trauma care are used as quality indicators in the ACS NSQIP program, we wondered whether time points that are registered in hospital databases are reliable enough to be used as such. We did not formulate an a priori assumption about the relevant difference between recordings since this is highly dependent on the specific purpose of the measurement the recordings are used for. In case of life-saving measures differences of minutes could be relevant while greater differences could be accepted in case of other research topics.

**Figure 2:**
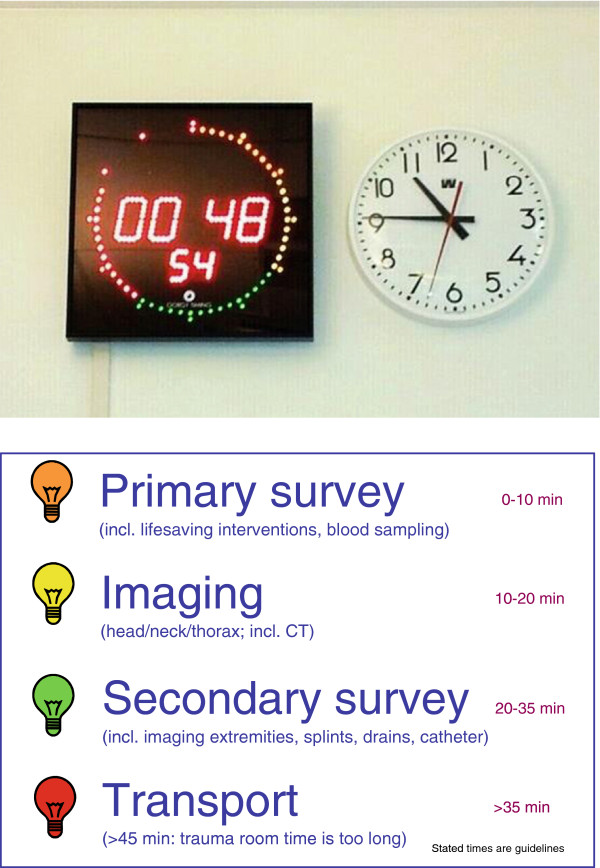
Trauma clock and adjoining poster.

Time intervals are useful as quality indicators when they reflect the efficiency of the provided trauma care. It should be fully clear that gaining time in trauma care should not be an aim in itself. Trauma care is suited to the unique needs of each patient and all medically indicated diagnostic and interventional procedures should be performed, regardless of the time it takes. This is reflected by our finding that patients with an ISS between 16 and 24 have the longest trauma room time: these patients are mostly haemodynamically stable enough to remain at the trauma room where central lines can be placed, tubes and drains can be inserted and most diagnostics can be realised. However, during the current economic challenging times in health care, efficient time management in the trauma room is desirable. This will make the trauma room available for new admissions and it will allow medical, nursing and other personnel involved to shift their attention (back) to other, more or less urgent patients, or other (non-clinical) duties.To raise the awareness of time management during trauma care in our hospital, a specially developed trauma clock is attached to a wall in the trauma room (Figure 2). The colours of the LED light in the outer circle represent time intervals relevant during trauma care and correspond with the adjoining poster. The following target time points were set up: the primary survey should be finished in 10 minutes (orange), another 10 minutes are needed to do radiologic imaging (yellow), the consecutive 15 minutes are used for secondary survey (green) and preferably, after 35 minutes a patient should be ready for transport (red). Although no formal research on this topic has been done yet, we have the impression that the clock raises the awareness of time management during trauma care. Especially young residents, for whom the learning experience of being the trauma team leader is demanding itself, mention that they are more aware of the time they spend in the trauma room with each patient. The Trauma Clock is currently being further refined and made commercially available (adjustments are possible according to local specifications and wishes).

Dedicatedly registered intervals might be preferred above routinely recorded time intervals when used as quality indicators, but this method is labour-intensive. An alternative is improving the routinely registered time intervals. This could be done by linking routinely recorded time intervals to routinely executed actions at the trauma room. A pressure plate in the entrance of the trauma room, that automatically records time of arrival, for example. An automatically recorded time of arrival when the patient is connected to the monitoring device is an inexpensive alternative. Besides registering time intervals dedicatedly or by linking routinely recorded time intervals to routinely executed actions there is a third option. This is the use of Real-Time Location Systems like radio-frequency identification (RFID) [15]. The way RFID works is simple. A small tag on a device or person emits a radio wave that is detected by a network of receivers around the hospital. Software states the position of the patient and puts the location into a hospital information system. The same software can link time intervals to the location. This creates a very accurate way of recording time intervals. Though it is expensive to build such an infrastructure, it can help the staff to work more efficiently by providing them with real-time information.

The main limitation of our study is the size of the patient sample and the subsequent relatively small absolute amount of multi trauma patients. Differences might be greater than we assume in a larger study population, although the characteristics of the study population are representative for trauma patients in our centre and included patients did not differ in baseline characteristics from excluded patients.

Another limitation is that the compared time intervals are both at least partially biased by human factors. Not all routinely recorded time intervals are therefore strictly ‘routinely recorded’, trauma room time and ICU time for example depend on human factors at least partially. However, our aim was to assess the reliability of time intervals as recorded in our hospital databases. These time intervals are used for research purposes and were therefore not corrected for bias in human factors. Furthermore, we could have validated the dedicated time registrations by a second independent observer or video recording. However, video recording might be even more subjective than ‘on-scene’ registrations, since not all actions might be visible. The independent researcher was not involved in trauma care and his only task was to register the time intervals thereby reducing the risk of bias.

Strength of our study is that it reflects daily practice. Most retrospective studies use routinely recorded time intervals under the assumption that these intervals correspond with the real intervals. To our knowledge, this is the first study on the topic of trauma patients that questions this assumption. Especially when time intervals are used as quality indicators, it is of major importance to know whether these time intervals are realistic enough to be judged on. Furthermore, we developed a device which can be useful in increasing the awareness of the passing of time during trauma work-up.

## Conclusion

This study shows that routinely recorded time intervals in the trauma care setting differ statistically significant from dedicatedly registered intervals.

## Competing interests

All the authors declare that there is no financial support or relationship that may pose conflict of interest.

## Authors’ contributions

JCS and EWMdJ conducted the research and wrote the article under the direct supervision of NWLS and JCG. All authors read and approved the final manuscript.
